# Synthesis, crystal structure and Hirshfeld surface analysis of ethyl (*E*)-2-cyano-3-[5-(4-ethyl­phen­yl)isoxazol-3-yl]prop-2-enoate

**DOI:** 10.1107/S2056989025006875

**Published:** 2025-08-19

**Authors:** Irina A. Kolesnik, Artem P. Sanchez-Pimentel, Mikhail S. Grigoriev, Tuncer Hökelek, Khudayar I. Hasanov, Narmina A. Guliyeva, Tahir A. Javadzade, Mohammed Hadi Al-Douh

**Affiliations:** a Institute of Physical Organic Chemistry of National Academy of Sciences of Belarus, 13 Surganov Str., 220072 Minsk, Belarus; b Faculty of Science RUDN University 6 Miklukho-Maklaya Str 117198 Moscow Russian Federation; c Frumkin Institute of Physical Chemistry and Electrochemistry, Russian Academy of Sciences, 31 Bldg 4, Leninsky prosp, 119071 Moscow, Russian Federation; d Hacettepe University, Department of Physics, 06800 Beytepe-Ankara, Türkiye; e Azerbaijan Medical University, Scientific Research Centre (SRC), A. Kasumzade Str. 14, AZ1022, Baku, Azerbaijan; f Baku Engineering University, Khirdalan, Hasan Aliyev Str. 120, AZ0101, Absheron, Azerbaijan; g Department of Chemistry and Chemical Engineering, Khazar University, Mahsati Str. 41, AZ1096, Baku, Azerbaijan; h Chemistry Department, Faculty of Science, Hadhramout University, Mukalla, Hadhramout, Yemen; Universidade de Sâo Paulo, Brazil

**Keywords:** crystal structure, isoxazole ring, cyano­acrylaate, Hirshfeld surface analysis

## Abstract

In the mol­ecule of the title compound, C_17_H_16_N_2_O_3_, the isoxazol and phenyl rings are oriented at a dihedral angle of 14.84 (5)°. The 2-cyano­acrylate moiety is in *E*- configuration. In the crystal, there are no inter­molecular hydrogen-bonding or C—H⋯π(ring) inter­actions, only a π–π inter­action between the parallel isoxazol rings with centroid-to-centroid distance of 3.4932 (9) Å (α = 0.02°). The Hirshfeld surface analysis indicates that the most important contributions to the crystal packing are from H⋯H (43.9%), H⋯N/N⋯H (17.0%), H⋯O/O⋯H (13.9%) and C⋯C (10.1%) inter­actions.

## Abstract 2

The asymmetric unit of the title compound contains isoxazol and phenyl rings. The 2-cyano­acrylate moiety is in an *E*- configuration. In crystal, there are there are no inter­molecular hydrogen-bonding or C—H⋯π(ring) inter­actions, only a π–π inter­action between the parallel isoxazol rings with centroid-to-centroid distance of 3.4932 (9) Å.

## 1. Chemical context

Esters of cyano­acrylic acid – cyano­acrylates – are well-known to be the components of adhesive compositions. They are also convenient and versatile building blocks in organic synthesis that are widely used in processes of carbon skeleton growth (Ammida & Giath, 2003 ▸; Kim *et al.*, 2005 ▸; Motokura *et al.*, 2019 ▸). They have found wide applications in the syntheses of cyclic and heterocyclic structures, including condensed derivatives. In particular, they are used to obtain polysubstituted cyclo­propanes (Zhang *et al.*, 2017 ▸), cyclo­hexenes (Xiao *et al.*, 2012 ▸), cyano­anilines (Sharanin *et al.*, 1980 ▸), chromenes (Dong *et al.*, 2010 ▸; Chang *et al.*, 2021 ▸), pyran­ocoumarines (Xie *et al.*, 2022 ▸), pyrrolidines (Imagawa *et al.*, 2016 ▸), tetra­hydro­furans (Khan *et al.*, 2014 ▸), pyrimidines (Moirangthem & Laitonjam, 2009 ▸; Sheibani *et al.*, 2009 ▸), piperidines (Dong *et al.*, 2021 ▸) and others. The most commonly used substrates are 3-aryl and 3-hetaryl 2-cyano­acrylates. As a result of the asymmetry of the spatial structure of the mol­ecule, they can exist in the form of *Z*- and *E*-isomers, which can ultimately affect the spatial structure of the products resulting from the reactions with their participation. The configuration of the tris­ubstituted double bond of cyano­acrylates depends on the synthesis conditions and structure of the substrates (Irfan *et al.*, 2021 ▸). This determines the importance of accurately establishing the crystal structure of cyano­acrylates. Herein, we report on the crystal structure of ethyl (*E*)-2-cyano-3-[5-(4-ethyl­phen­yl)isoxazol-3-yl]prop-2-enoate (**1**), which we studied as a starting compound for the synthesis of isoxazole-containing pyrans and chromenes (Potkin *et al.*, 2024 ▸). According to literature data, similar in structure aryl and hetaryl cyano­acrylates also have the *E*-configuration of the exocyclic double bond (Deshpande *et al.*, 2012 ▸; Franconetti *et al.*, 2016 ▸; Kalkhambkar *et al.*, 2012 ▸).
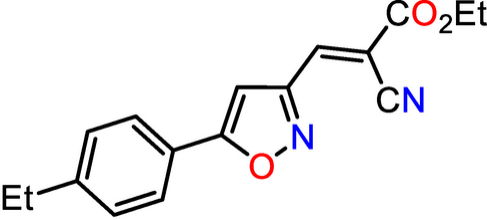


## 2. Structural commentary

**Figure 1:**
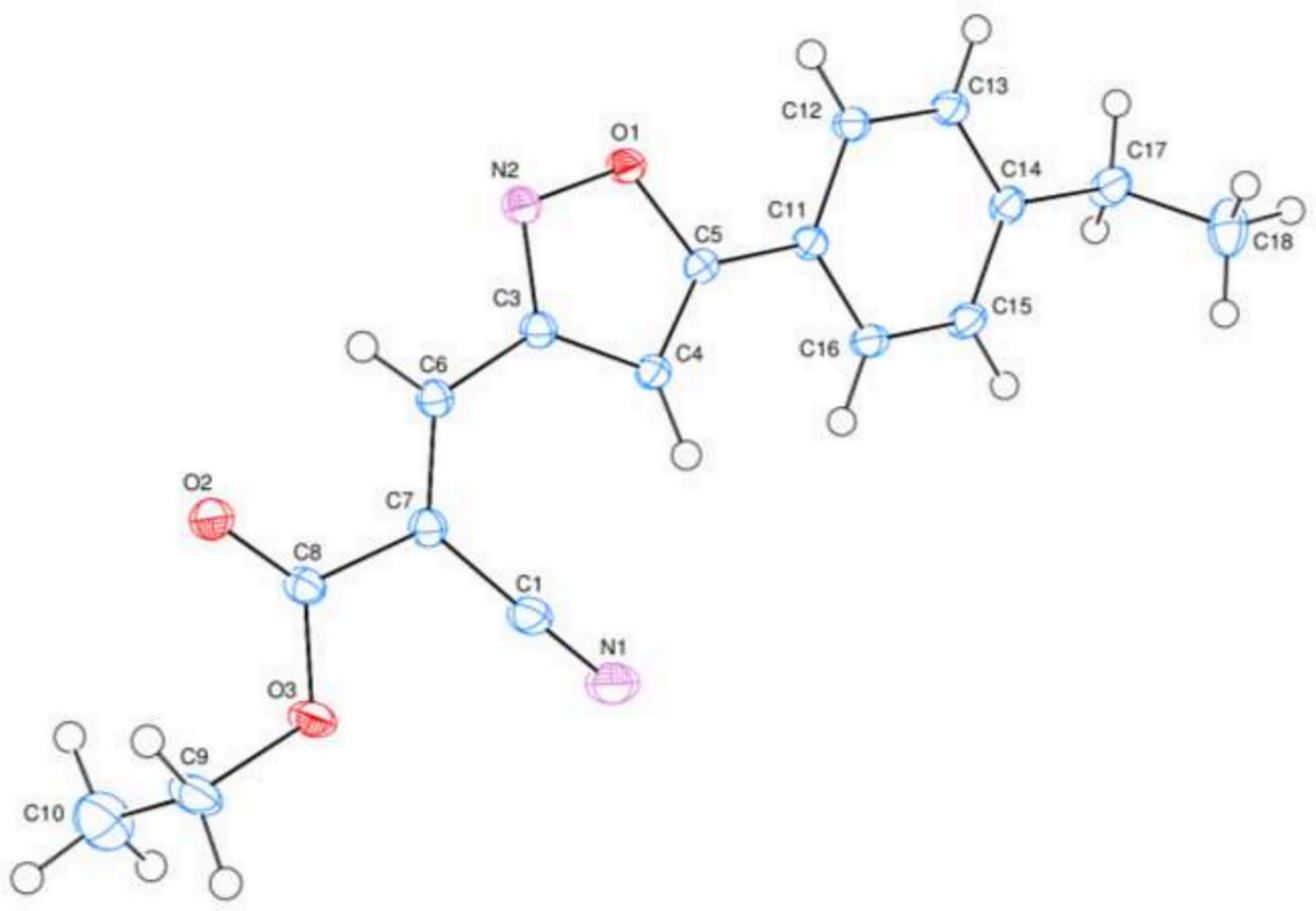
The asymmetric unit of the title compound with atom-numbering scheme and 50% probability ellipsoids.

The asymmetric unit of the title compound, C_17_H_16_N_2_O_3_, contains isoxazol (O1/N2/C3–C5) and phenyl (C11–C16) rings oriented at a dihedral angle of 14.84 (5)° (Fig. 1 ▸). The corresponding dihedral angles are reported to be 46.22 (4)° in C_17_H_13_NO_3_ (Benouatas *et al.*, 2021 ▸) and 5.50 (8)° in C_17_H_13_NO_2_ (Asiri *et al.*, 2012 ▸). Atoms C1, C6, C7, N1 and C17 are located 0.0178 (18), 0.0389 (15), 0.0641 (15), −0.044 (2) and −0.0429 (15) Å, respectively, away from the corresponding ring planes. In the ethyl 2-cyano­acrylate moiety, the C9—O3—C8—C7, O3—C8—C7—C6, C8—C7—C6—C3 and C7—C6—C3—N2 torsion angles are −179.66 (12), 175.16 (13), −177.75 (12) and 179.15 (14)°, respectively, showing an *E*-configuration. Bond lengths and angles agree well with the values observed for the related compounds (*Z*)-4-(2-meth­oxy­benzyl­idene)-3-phenyl­isoxazol-5(4*H*)-one (Ben­ouatas *et al.*, 2021 ▸) and (4*Z*)-4-benzyl­idene-2-phenyl-1,3-oxazol-5(*4*H)-one (Asiri *et al.*, 2012 ▸).

## 3. Supra­molecular features

**Figure 2:**
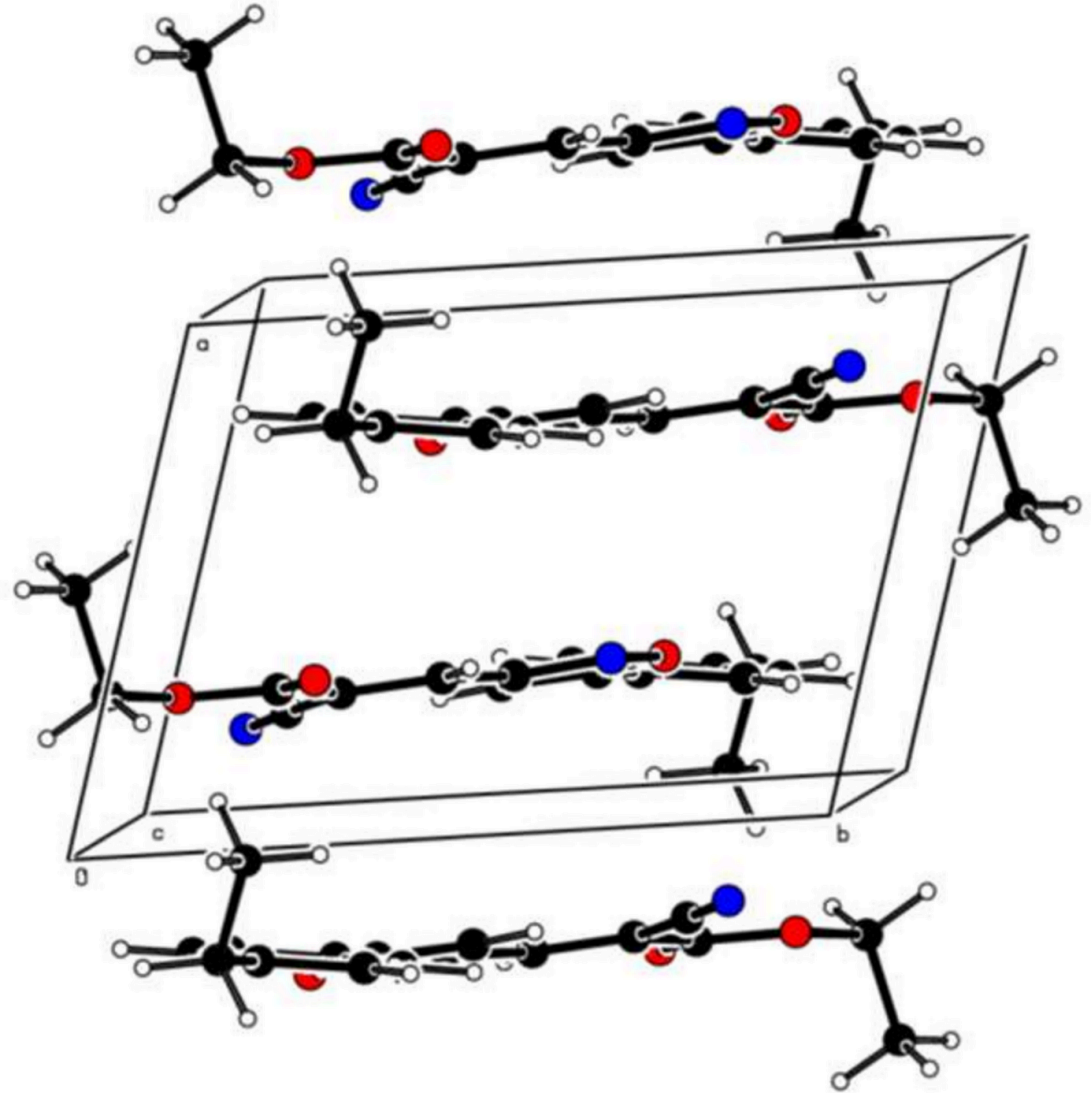
A partial packing diagram for the title compound viewed down the *c*-axis direction.

In the crystal, the mol­ecules are stacked along the *a*-axis direction (Fig. 2 ▸) and there are no inter­molecular hydrogen-bonding or C—H⋯π(ring) inter­actions, only a π–π inter­action between the parallel isoxazol (O1/N2/C3–C5) rings with a centroid-to-centroid distance of 3.4932 (9) Å (α = 0.02°).

## 4. Hirshfeld surface analysis

**Figure 3:**
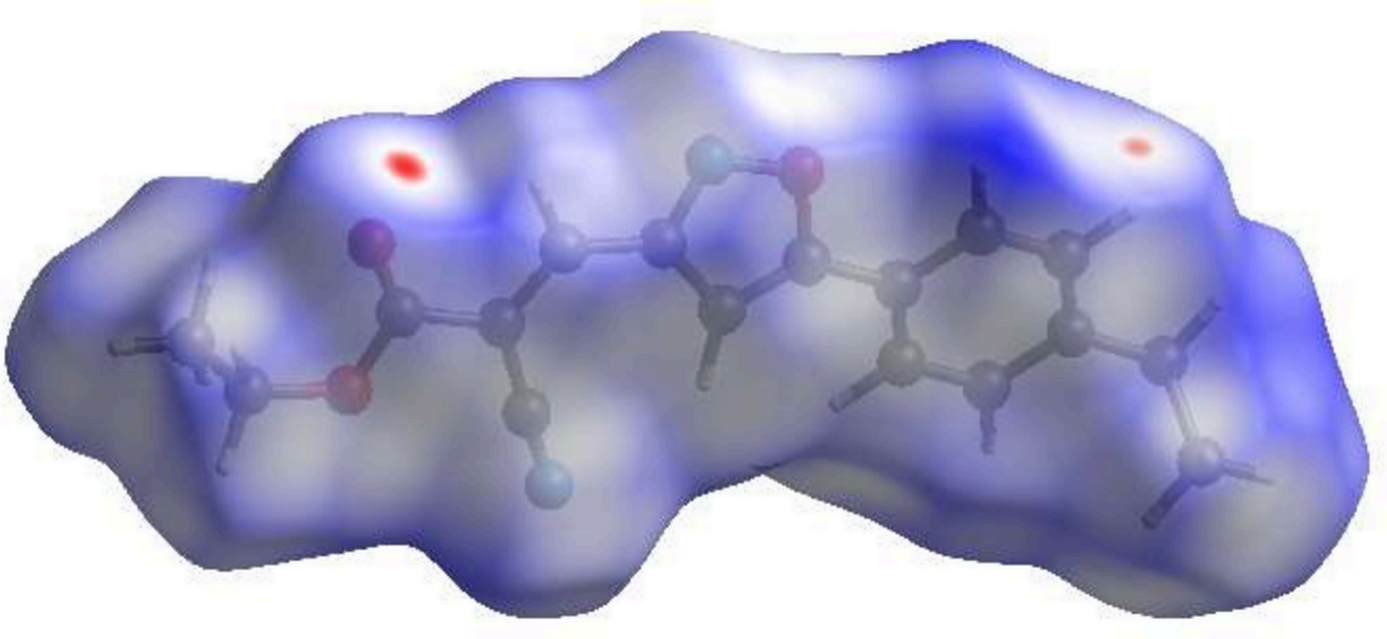
View of the three-dimensional Hirshfeld surface plotted over *d*_norm_.

**Figure 4:**
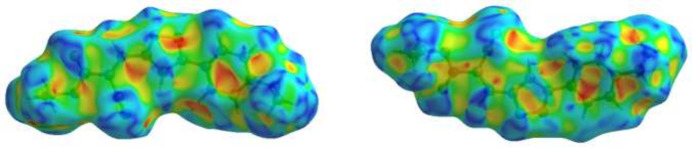
The shape-index surface showing two orientations.

**Figure 5:**
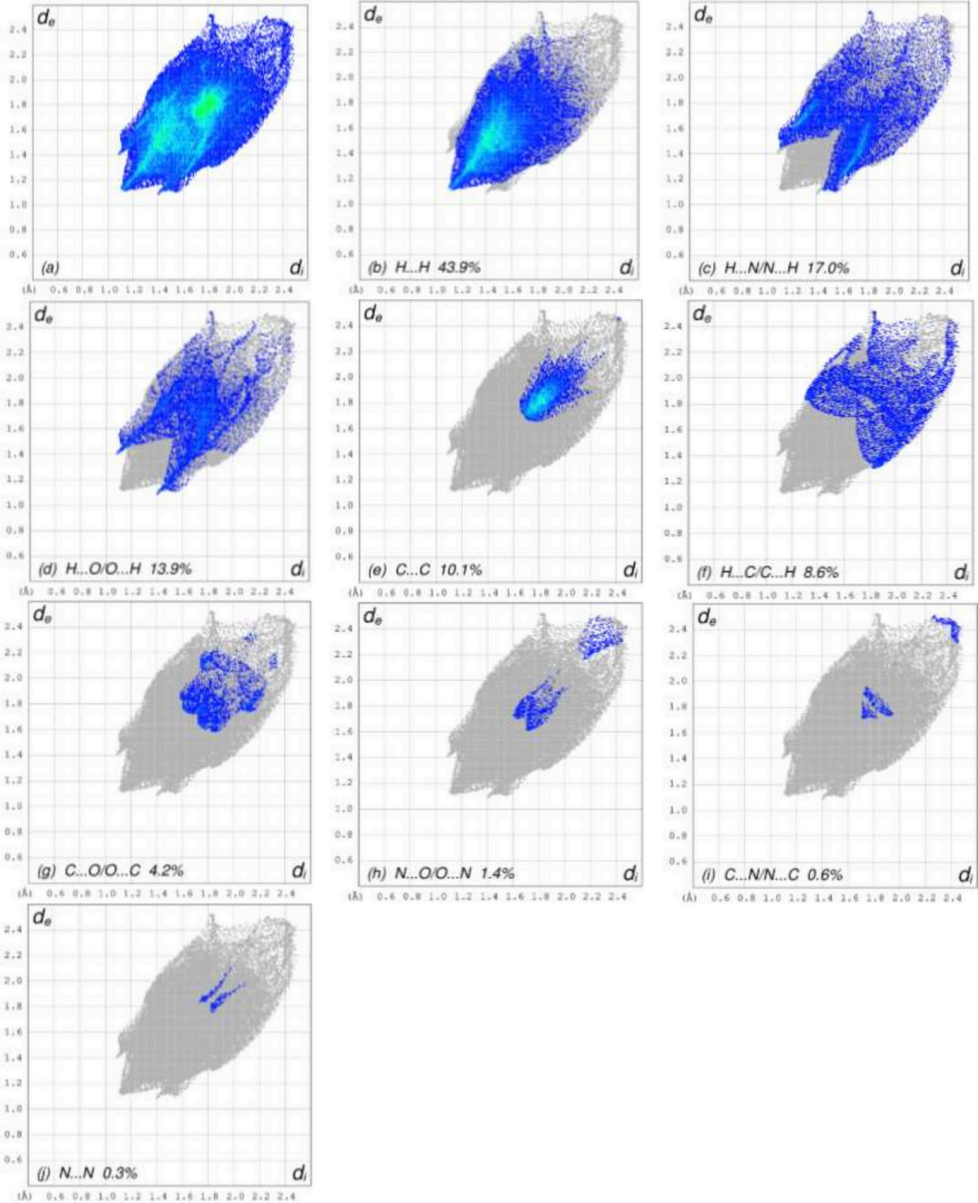
The full two-dimensional fingerprint plots showing (*a*) all inter­actions, and delineated into (*b*) H⋯H, (*c*) H⋯N/N⋯H, (*d*) H⋯O/O⋯H, (*e*) C⋯C, (*f*) H⋯C/C⋯H, (*g*) C⋯O/O⋯C, (*h*) N⋯O/O⋯N, (*i)* C⋯N/N⋯C and (*j*) N⋯N inter­actions. The *d*_i_ and *d*_e_ values are the closest inter­nal and external distances (in Å) from given points on the Hirshfeld surface.

To visualize the inter­molecular inter­actions in the crystal of title compound, a Hirshfeld surface (HS) analysis was carried out using *Crystal Explorer 17.5* (Spackman *et al.*, 2021 ▸). In the HS plotted over *d*_norm_ (Fig. 3 ▸), the white surface indicates contacts with distances equal to the sum of van der Waals radii, and the red and blue colours indicate distances shorter (in close contact) or longer (distinct contact) than the van der Waals radii, respectively (Table 1 ▸) (Venkatesan *et al.*, 2016 ▸). The appearing bright-red spots indicate their roles as the respective donors and/or acceptors. The shape-index surface can be used to identify characteristic packing modes, in particular, planar stacking arrangements and the presence of aromatic stacking inter­actions such as C—H⋯π and π–π inter­actions with the former represented as red π-holes, which are related to the electron ring inter­actions between the C–H groups with the centroid of the aromatic rings of neighbouring mol­ecules. Fig. 4 ▸ clearly suggests that there are no C—H⋯π inter­actions. π–π stacking is indicated by the presence of adjacent red and blue triangles; if there are no adjacent red and/or blue triangles, then there are no π–π inter­actions. Fig. 4 ▸ clearly suggests that there are π⋯π inter­actions in the title compound. According to the two-dimensional fingerprint plots (Fig. 5 ▸), H⋯H (43.9%), H⋯N/N⋯H (17.0%), H⋯O/O⋯H (13.9%) and C⋯C (10.1%) contacts make the most important contributions to the HS.

## 5. Synthesis and crystallization

**Figure 6:**
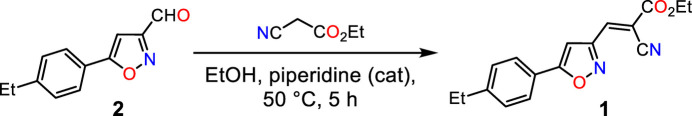
Reaction scheme.

Compound **1** was obtained according to the method (Fig. 6 ▸) described by us earlier (Potkin *et al.*, 2024 ▸). 5-(4-Ethyl­phen­yl)isoxazole-3-carbaldehyde (**2**) (0.50 g, 2.5 mmol) and ethyl cyano­acetate (0.34 g, 3.0 mmol) were dissolved in EtOH (10 ml), 2 drops of piperidine were added and the resulting mixture was stirred for 5 h at 323 K then kept at 278 K overnight. The resulting precipitate was filtered, washed with cold EtOH (2 × 5 ml) and dried under reduced pressure. The obtained product did not require further purification. It was crystallized from methanol solution to give pale creamy needles, yield 0.50 g (67%), m.p. 274–276 K. IR (KBr), ν (cm^−1^): 2232 (CN), 1737 (C=O), 1663, 1613, 1592, 1561 (1,2-oxazole), 1245 (aromatic C–H), 797 (vinyl­ene –CH=), 534 (–CH=C—CN). ^1^H NMR (CDCl_3_, 500 MHz, 301 K): δ = 7.95 (*s*, 1H, –CH=), 7.75 (*d*, 2H, HAr, *J* = 8.4), 7.37 (*s*, 1H, H4), 7.32 (*d*, 2H, H Ar, *J* = 8.4), 4.42 (*q*, 2H, CH_2_, *J* = 7.1), 2.71 (*q*, 2H, CH_2_, *J* = 7.6), 1.41 (*t*, 3H, CH_3_, *J* = 7.1), 1.27 (*t*, 3H, CH_3_, *J* = 7.6). ^13^C NMR (CDCl_3_, 125 MHz, 301 K): δ = 172.6, 161.0, 157.9, 147.9, 142.8, 128.8 (2C), 124.0, 114.2, 109.8, 97.6, 63.5, 29.0, 15.4, 14.2. MS (APCI): *m*/*z* = 296 [*M*]^+^ (100).

## 6. Refinement

Crystal data, data collection and structure refinement details are summarized in Table 2 ▸. The C-bound hydrogen-atom positions were calculated geometrically at distances of 0.95 Å (for aromatic CH), 0.99 Å (for CH_2_) and 0.98 Å (for CH_3_), and refined using a riding model applying the constraint *U*_iso_ = *k* × *U*_eq_ (C), where *k* = 1.5 for methyl hydrogens and *k* = 1.2 for all other hydrogen atoms.

## Supplementary Material

Crystal structure: contains datablock(s) I. DOI: 10.1107/S2056989025006875/ex2093sup1.cif

Structure factors: contains datablock(s) I. DOI: 10.1107/S2056989025006875/ex2093Isup2.hkl

Supporting information file. DOI: 10.1107/S2056989025006875/ex2093Isup3.cml

CCDC reference: 2477570

Additional supporting information:  crystallographic information; 3D view; checkCIF report

## Figures and Tables

**Table 1:** Selected interatomic distances (Å)

O1⋯H12	2.52	C1⋯C4	3.069 (2)
O1⋯H9*A*^i^	2.66	C3⋯C11^iv^	3.403 (2)
O2⋯H9*B*	2.38	C6⋯C11^iv^	3.385 (2)
O2⋯H15^ii^	2.61	C6⋯C16^iv^	3.404 (2)
O2⋯H6	2.48	C1⋯H4	2.63
N1⋯H13^iii^	2.65	C4⋯H16	2.82
N1⋯H4	2.65	H13⋯H17*A*	2.37
N2⋯H9*A*^i^	2.69		

Symmetry codes: (i) 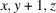; (ii) 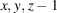; (iii) 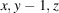; (iv) 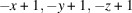.

**Table 2:** Experimental details

Crystal data	
Chemical formula	C_17_H_16_N_2_O_3_
*M*_r_	296.32
Crystal system, space group	Triclinic, *P* 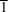
Temperature (K)	100
*a*, *b*, *c* (Å)	7.2361 (6), 10.1537 (8), 11.1493 (8)
α, β, γ (°)	73.098 (2), 76.611 (3), 72.555 (3)
*V* (Å^3^)	738.32 (10)
*Z*	2
Radiation type	Mo *K*α
μ (mm^−1^)	0.09
Crystal size (mm)	0.40 × 0.32 × 0.20

Data collection	
Diffractometer	Bruker Kappa APEXII
Absorption correction	Multi-scan (*SADABS*; Krause et al., 2015 ▸)
*T*_min_, *T*_max_	0.930, 1.000
No. of measured, independent and observed [*I* > 2σ(*I*)] reflections	13453, 4283, 3544
*R*_int_	0.020
(sin θ/λ)_max_ (Å^−1^)	0.703

Refinement	
*R*[*F*^2^ > 2σ(*F*^2^)], *wR*(*F*^2^), *S*	0.055, 0.150, 1.02
No. of reflections	4283
No. of parameters	201
H-atom treatment	H-atom parameters constrained
Δρ_max_, Δρ_min_ (e Å^−3^)	0.74, −0.62

Computer programs: *APEX3* and *SAINT* (Bruker, 2014 ▸), *SHELXT2019/1* (Sheldrick, 2015*a* ▸), *SHELXL2018/3* (Sheldrick, 2015*b* ▸) and *SHELXTL* (Sheldrick, 2008 ▸).

## supplementary crystallographic information

### Ethyl (E)-2-cyano-3-[5-(4-ethylphenyl)isoxazol-3-yl]prop-2-enoate Crystal data

**Table:**

C_17_H_16_N_2_O_3_	*Z* = 2
*M**_r_* = 296.32	*F*(000) = 312
Triclinic, *P*1	*D*_x_ = 1.333 Mg m^−^^3^
*a* = 7.2361 (6) Å	Mo *K*α radiation, λ = 0.71073 Å
*b* = 10.1537 (8) Å	Cell parameters from 5551 reflections
*c* = 11.1493 (8) Å	θ = 2.5–30.1°
α = 73.098 (2)°	µ = 0.09 mm^−^^1^
β = 76.611 (3)°	*T* = 100 K
γ = 72.555 (3)°	Bulk, colourless
*V* = 738.32 (10) Å^3^	0.40 × 0.32 × 0.20 mm

### Ethyl (E)-2-cyano-3-[5-(4-ethylphenyl)isoxazol-3-yl]prop-2-enoate Data collection

**Table:**

Bruker Kappa APEXII diffractometer	3544 reflections with *I* > 2σ(*I*)
φ and ω scans	*R*_int_ = 0.020
Absorption correction: multi-scan (SADABS; Krause et al., 2015)	θ_max_ = 30.0°, θ_min_ = 3.8°
*T*_min_ = 0.930, *T*_max_ = 1.000	*h* = −8→10
13453 measured reflections	*k* = −14→14
4283 independent reflections	*l* = −15→15

### Ethyl (E)-2-cyano-3-[5-(4-ethylphenyl)isoxazol-3-yl]prop-2-enoate Refinement

**Table:**

Refinement on *F*^2^	0 restraints
Least-squares matrix: full	Hydrogen site location: inferred from neighbouring sites
*R*[*F*^2^ > 2σ(*F*^2^)] = 0.055	H-atom parameters constrained
*wR*(*F*^2^) = 0.150	*w* = 1/[σ^2^(*F*_o_^2^) + (0.073*P*)^2^ + 0.5251*P*] where *P* = (*F*_o_^2^ + 2*F*_c_^2^)/3
*S* = 1.02	(Δ/σ)_max_ < 0.001
4283 reflections	Δρ_max_ = 0.74 e Å^−^^3^
201 parameters	Δρ_min_ = −0.62 e Å^−^^3^

### Ethyl (E)-2-cyano-3-[5-(4-ethylphenyl)isoxazol-3-yl]prop-2-enoate Special details

**Table:**

Geometry. All esds (except the esd in the dihedral angle between two l.s. planes) are estimated using the full covariance matrix. The cell esds are taken into account individually in the estimation of esds in distances, angles and torsion angles; correlations between esds in cell parameters are only used when they are defined by crystal symmetry. An approximate (isotropic) treatment of cell esds is used for estimating esds involving l.s. planes.

### Ethyl (E)-2-cyano-3-[5-(4-ethylphenyl)isoxazol-3-yl]prop-2-enoate Fractional atomic coordinates and isotropic or equivalent isotropic displacement parameters (Å^2^)

**Table:**

	*x*	*y*	*z*	*U*_iso_*/*U*_eq_	
O1	0.28675 (16)	0.69298 (11)	0.44138 (9)	0.0194 (2)	
O2	0.30302 (17)	0.26261 (11)	0.13160 (10)	0.0219 (2)	
O3	0.27135 (18)	0.07239 (11)	0.29464 (10)	0.0234 (2)	
N1	0.1827 (3)	0.14647 (16)	0.57881 (14)	0.0370 (4)	
N2	0.2963 (2)	0.63165 (13)	0.34260 (11)	0.0200 (3)	
C1	0.2185 (2)	0.20516 (16)	0.47481 (14)	0.0229 (3)	
C3	0.26948 (19)	0.50293 (14)	0.39795 (13)	0.0152 (3)	
C4	0.24135 (19)	0.47687 (14)	0.53287 (13)	0.0154 (3)	
H4	0.219017	0.393425	0.593852	0.018*	
C5	0.25385 (19)	0.59890 (14)	0.55416 (13)	0.0148 (3)	
C6	0.27883 (19)	0.41529 (14)	0.31311 (13)	0.0156 (3)	
H6	0.301540	0.457438	0.224765	0.019*	
C7	0.2590 (2)	0.28166 (14)	0.34550 (13)	0.0159 (3)	
C8	0.2809 (2)	0.20658 (14)	0.24319 (13)	0.0166 (3)	
C9	0.2914 (3)	−0.01480 (16)	0.20686 (16)	0.0273 (3)	
H9A	0.212681	−0.085624	0.245866	0.033*	
H9B	0.241248	0.046024	0.128222	0.033*	
C10	0.4965 (3)	−0.0876 (2)	0.1760 (2)	0.0374 (4)	
H10A	0.572276	−0.017378	0.130783	0.056*	
H10B	0.507173	−0.151209	0.121968	0.056*	
H10C	0.548105	−0.143216	0.254400	0.056*	
C11	0.24115 (19)	0.64417 (14)	0.66934 (12)	0.0147 (3)	
C12	0.2177 (2)	0.78675 (15)	0.66627 (13)	0.0165 (3)	
H12	0.208358	0.856744	0.588440	0.020*	
C13	0.2081 (2)	0.82569 (15)	0.77770 (13)	0.0180 (3)	
H13	0.192322	0.922782	0.774977	0.022*	
C14	0.2212 (2)	0.72541 (15)	0.89328 (13)	0.0181 (3)	
C15	0.2458 (2)	0.58249 (16)	0.89528 (13)	0.0198 (3)	
H15	0.255225	0.512582	0.973156	0.024*	
C16	0.2564 (2)	0.54221 (15)	0.78484 (13)	0.0178 (3)	
H16	0.274169	0.444908	0.787414	0.021*	
C17	0.2048 (3)	0.76846 (17)	1.01452 (14)	0.0244 (3)	
H17A	0.213608	0.868262	0.993749	0.029*	
H17B	0.316062	0.708018	1.058750	0.029*	
C18	0.0129 (3)	0.7546 (2)	1.10285 (16)	0.0313 (4)	
H18A	−0.097706	0.812084	1.058595	0.047*	
H18B	0.005407	0.788052	1.178329	0.047*	
H18C	0.007697	0.654765	1.128448	0.047*	

### Ethyl (E)-2-cyano-3-[5-(4-ethylphenyl)isoxazol-3-yl]prop-2-enoate Atomic displacement parameters (Å^2^)

**Table:**

	*U*^11^	*U*^22^	*U*^33^	*U*^12^	*U*^13^	*U*^23^
O1	0.0318 (6)	0.0145 (5)	0.0139 (5)	−0.0080 (4)	−0.0037 (4)	−0.0041 (4)
O2	0.0335 (6)	0.0191 (5)	0.0158 (5)	−0.0097 (4)	−0.0050 (4)	−0.0038 (4)
O3	0.0413 (6)	0.0133 (5)	0.0183 (5)	−0.0077 (4)	−0.0089 (4)	−0.0034 (4)
N1	0.0710 (12)	0.0230 (7)	0.0202 (7)	−0.0198 (7)	−0.0045 (7)	−0.0038 (5)
N2	0.0323 (7)	0.0165 (6)	0.0140 (5)	−0.0083 (5)	−0.0042 (5)	−0.0052 (4)
C1	0.0364 (8)	0.0162 (6)	0.0184 (7)	−0.0089 (6)	−0.0048 (6)	−0.0050 (5)
C3	0.0167 (6)	0.0147 (6)	0.0147 (6)	−0.0038 (4)	−0.0032 (4)	−0.0036 (5)
C4	0.0185 (6)	0.0147 (6)	0.0139 (6)	−0.0060 (5)	−0.0017 (5)	−0.0037 (5)
C5	0.0160 (6)	0.0142 (6)	0.0137 (6)	−0.0041 (4)	−0.0020 (4)	−0.0026 (5)
C6	0.0176 (6)	0.0153 (6)	0.0146 (6)	−0.0043 (5)	−0.0030 (5)	−0.0041 (5)
C7	0.0184 (6)	0.0159 (6)	0.0147 (6)	−0.0045 (5)	−0.0037 (5)	−0.0043 (5)
C8	0.0197 (6)	0.0138 (6)	0.0176 (6)	−0.0038 (5)	−0.0051 (5)	−0.0045 (5)
C9	0.0466 (10)	0.0154 (7)	0.0241 (7)	−0.0066 (6)	−0.0131 (7)	−0.0067 (6)
C10	0.0396 (10)	0.0355 (10)	0.0414 (10)	−0.0102 (8)	−0.0068 (8)	−0.0145 (8)
C11	0.0158 (6)	0.0149 (6)	0.0148 (6)	−0.0053 (4)	−0.0025 (4)	−0.0041 (5)
C12	0.0198 (6)	0.0146 (6)	0.0156 (6)	−0.0053 (5)	−0.0034 (5)	−0.0029 (5)
C13	0.0235 (6)	0.0155 (6)	0.0171 (6)	−0.0079 (5)	−0.0019 (5)	−0.0047 (5)
C14	0.0224 (6)	0.0202 (7)	0.0147 (6)	−0.0101 (5)	−0.0008 (5)	−0.0054 (5)
C15	0.0269 (7)	0.0190 (6)	0.0139 (6)	−0.0093 (5)	−0.0028 (5)	−0.0011 (5)
C16	0.0225 (6)	0.0148 (6)	0.0167 (6)	−0.0072 (5)	−0.0024 (5)	−0.0026 (5)
C17	0.0389 (8)	0.0253 (7)	0.0151 (6)	−0.0173 (6)	−0.0023 (6)	−0.0057 (5)
C18	0.0311 (8)	0.0440 (10)	0.0187 (7)	−0.0055 (7)	−0.0023 (6)	−0.0126 (7)

### Ethyl (E)-2-cyano-3-[5-(4-ethylphenyl)isoxazol-3-yl]prop-2-enoate Geometric parameters (Å, º)

**Table:**

O1—C5	1.3600 (16)	C10—H10B	0.9800
O1—N2	1.3940 (15)	C10—H10C	0.9800
O2—C8	1.2033 (17)	C11—C12	1.3973 (18)
O3—C8	1.3319 (17)	C11—C16	1.4016 (18)
O3—C9	1.4573 (18)	C12—C13	1.3907 (19)
N1—C1	1.148 (2)	C12—H12	0.9500
N2—C3	1.3244 (18)	C13—C14	1.3935 (19)
C1—C7	1.434 (2)	C13—H13	0.9500
C3—C4	1.4260 (18)	C14—C15	1.402 (2)
C3—C6	1.4542 (18)	C14—C17	1.507 (2)
C4—C5	1.3594 (18)	C15—C16	1.385 (2)
C4—H4	0.9500	C15—H15	0.9500
C5—C11	1.4602 (18)	C16—H16	0.9500
C6—C7	1.3417 (19)	C17—C18	1.523 (2)
C6—H6	0.9500	C17—H17A	0.9900
C7—C8	1.5023 (19)	C17—H17B	0.9900
C9—C10	1.456 (3)	C18—H18A	0.9800
C9—H9A	0.9900	C18—H18B	0.9800
C9—H9B	0.9900	C18—H18C	0.9800
C10—H10A	0.9800		
			
O1···H12	2.52	C1···C4	3.069 (2)
O1···H9A^i^	2.66	C3···C11^iv^	3.403 (2)
O2···H9B	2.38	C6···C11^iv^	3.385 (2)
O2···H15^ii^	2.61	C6···C16^iv^	3.404 (2)
O2···H6	2.48	C1···H4	2.63
N1···H13^iii^	2.65	C4···H16	2.82
N1···H4	2.65	H13···H17A	2.37
N2···H9A^i^	2.69		
			
C5—O1—N2	109.17 (10)	H10A—C10—H10C	109.5
C8—O3—C9	116.59 (12)	H10B—C10—H10C	109.5
C3—N2—O1	105.59 (11)	C12—C11—C16	119.36 (12)
N1—C1—C7	178.45 (18)	C12—C11—C5	121.30 (12)
N2—C3—C4	111.53 (12)	C16—C11—C5	119.33 (12)
N2—C3—C6	115.97 (12)	C13—C12—C11	119.68 (13)
C4—C3—C6	132.48 (13)	C13—C12—H12	120.2
C5—C4—C3	104.09 (12)	C11—C12—H12	120.2
C5—C4—H4	128.0	C12—C13—C14	121.44 (13)
C3—C4—H4	128.0	C12—C13—H13	119.3
C4—C5—O1	109.62 (12)	C14—C13—H13	119.3
C4—C5—C11	133.27 (12)	C13—C14—C15	118.46 (13)
O1—C5—C11	117.11 (11)	C13—C14—C17	121.33 (13)
C7—C6—C3	127.35 (13)	C15—C14—C17	120.20 (13)
C7—C6—H6	116.3	C16—C15—C14	120.67 (13)
C3—C6—H6	116.3	C16—C15—H15	119.7
C6—C7—C1	122.94 (13)	C14—C15—H15	119.7
C6—C7—C8	119.39 (12)	C15—C16—C11	120.38 (13)
C1—C7—C8	117.67 (12)	C15—C16—H16	119.8
O2—C8—O3	126.28 (13)	C11—C16—H16	119.8
O2—C8—C7	123.60 (12)	C14—C17—C18	112.18 (13)
O3—C8—C7	110.12 (12)	C14—C17—H17A	109.2
C10—C9—O3	110.18 (14)	C18—C17—H17A	109.2
C10—C9—H9A	109.6	C14—C17—H17B	109.2
O3—C9—H9A	109.6	C18—C17—H17B	109.2
C10—C9—H9B	109.6	H17A—C17—H17B	107.9
O3—C9—H9B	109.6	C17—C18—H18A	109.5
H9A—C9—H9B	108.1	C17—C18—H18B	109.5
C9—C10—H10A	109.5	H18A—C18—H18B	109.5
C9—C10—H10B	109.5	C17—C18—H18C	109.5
H10A—C10—H10B	109.5	H18A—C18—H18C	109.5
C9—C10—H10C	109.5	H18B—C18—H18C	109.5
			
C5—O1—N2—C3	−0.13 (15)	C1—C7—C8—O3	−4.46 (18)
O1—N2—C3—C4	0.20 (15)	C8—O3—C9—C10	91.16 (17)
O1—N2—C3—C6	−178.31 (11)	C4—C5—C11—C12	−166.21 (15)
N2—C3—C4—C5	−0.19 (16)	O1—C5—C11—C12	14.54 (19)
C6—C3—C4—C5	177.99 (14)	C4—C5—C11—C16	15.0 (2)
C3—C4—C5—O1	0.10 (15)	O1—C5—C11—C16	−164.26 (12)
C3—C4—C5—C11	−179.19 (14)	C16—C11—C12—C13	−0.6 (2)
N2—O1—C5—C4	0.02 (15)	C5—C11—C12—C13	−179.36 (12)
N2—O1—C5—C11	179.44 (11)	C11—C12—C13—C14	−0.1 (2)
N2—C3—C6—C7	179.15 (14)	C12—C13—C14—C15	0.4 (2)
C4—C3—C6—C7	1.0 (2)	C12—C13—C14—C17	−178.16 (13)
C3—C6—C7—C1	1.9 (2)	C13—C14—C15—C16	−0.1 (2)
C3—C6—C7—C8	−177.75 (12)	C17—C14—C15—C16	178.47 (14)
C9—O3—C8—O2	0.6 (2)	C14—C15—C16—C11	−0.5 (2)
C9—O3—C8—C7	−179.66 (12)	C12—C11—C16—C15	0.9 (2)
C6—C7—C8—O2	−5.0 (2)	C5—C11—C16—C15	179.68 (13)
C1—C7—C8—O2	175.34 (14)	C13—C14—C17—C18	109.02 (16)
C6—C7—C8—O3	175.16 (13)	C15—C14—C17—C18	−69.51 (19)

Symmetry codes: (i) *x*, *y*+1, *z*; (ii) *x*, *y*, *z*−1; (iii) *x*, *y*−1, *z*; (iv) −*x*+1, −*y*+1, −*z*+1.
